# Exudative retinopathy from retinal vasoproliferative tumor refractory to aflibercept treated with faricimab-svoa

**DOI:** 10.1016/j.ajoc.2026.102593

**Published:** 2026-05-07

**Authors:** Naveen Karthik, Syeda Fatima Abid, Yuxi Zheng, Miguel Materin, Xi Chen

**Affiliations:** a Duke Eye Center, 2351 Erwin Rd, Durham, NC, 27705, USA; b King Edward Medical University, Neelagumbad, Anarkali, Lahore, 54000, Pakistan; c Northwell Health, Manhattan Eye, Ear & Throat Hospital, New York, NY, 10065, USA

**Keywords:** Retinal vasoproliferative tumor, Faricimab-svoa, Aflibercept, Exudative retinal detachment, Exudative retinopathy

## Abstract

**Purpose:**

To report a case of exudative retinopathy secondary to retinal vasoproliferative tumor (RVPT) refractory to aflibercept treated with faricimab-svoa (faricimab).

**Principal results:**

A 43-year-old female presented with an exudative and hemorrhagic lesion in the periphery of the right eye consistent with RVPT. She was treated initially with three aflibercept injections every 4-6 weeks. Despite treatment, her exam worsened with increased exudation and subretinal fluid. She was then treated with five monthly faricimab injections, which resulted in resolution of subretinal fluid and exudates, and near complete regression of the RVPT. The patient subsequently developed a retinal tear at the base of the RVPT that was successfully treated with laser retinopexy. At one year follow-up, the RVPT remained fully regressed. She was monitored off therapy. No adverse event was noted.

**Conclusions:**

Faricimab has potential utility in treating exudative retinopathy secondary to RVPT. Further investigation with treatment trials and long-term follow-up is warranted.

## 1 Introduction

Retinal vasoproliferative tumors (RVPTs) are rare, benign retinal lesions characterized by abnormal vascular proliferation and are often associated with significant exudative complications, including macular edema, epiretinal membrane, subretinal exudates and fluid accumulation, and secondary retinal detachment.^1^ They may arise idiopathically or secondary to uveitis, retinitis pigmentosa, or Coats’ disease.^2^ Retinal structural changes associated with RVPTs include formation of telangiectatic vessels with hemorrhage and sub- and intraretinal exudates, which can lead to progressive vision loss.^1^

Treatment criteria for RVPTs include subretinal fluid (SRF) and/or hard exudates near the macula, cystoid macular edema (CME), and the presence of epiretinal membrane.^3^ Conventional management strategies include cryotherapy, laser photocoagulation, and radiotherapy.^4^ Recently, pharmacologic interventions such as intravitreal anti-vascular endothelial growth factor (VEGF) agents have been explored, primarily to manage secondary exudation and macular edema. Bevacizumab, ranibizumab, and aflibercept have shown modest success in prior studies with regressing the RVPT and associated edema and exudation; however, intravitreal injections with these agents are often insufficient as monotherapy and long-term outcomes are variable.^5^, ^6^, ^7^, ^8^.

Faricimab-svoa (faricimab), a bispecific monoclonal antibody that targets both VEGF-A and angiopoietin-2 (Ang-2) pathways, is an emerging therapeutic option for exudative retinopathy.^9^^,^^10^ This dual inhibition approach stabilizes vascular integrity and has demonstrated efficacy in neovascular age-related macular degeneration (nAMD) and diabetic macular edema (DME) as a primary or secondary therapy.^11^ Despite the broadening clinical use of faricimab, there are currently no published reports describing the application of faricimab in RVPTs. We present a case of a patient with exudative retinopathy secondary to RVPT who was treated successfully with intravitreal faricimab after worsening exudation despite intravitreal aflibercept.

## 2 Case presentation

A 43-year-old female presented to the ocular oncology clinic for evaluation of “white spots” in her peripheral vision of the right eye for several weeks, floaters, but no photopsias or visual field defects. She had no prior ocular history. She was seen by medical retina previously and treated with one injection of aflibercept for the possible diagnosis of a peripheral retinal cavernous hemangioma versus retinal hemangioblastoma of her right eye. Her past medical history was significant for ductal carcinoma in situ of the left breast and Lynch syndrome.

**Fig. 1:**
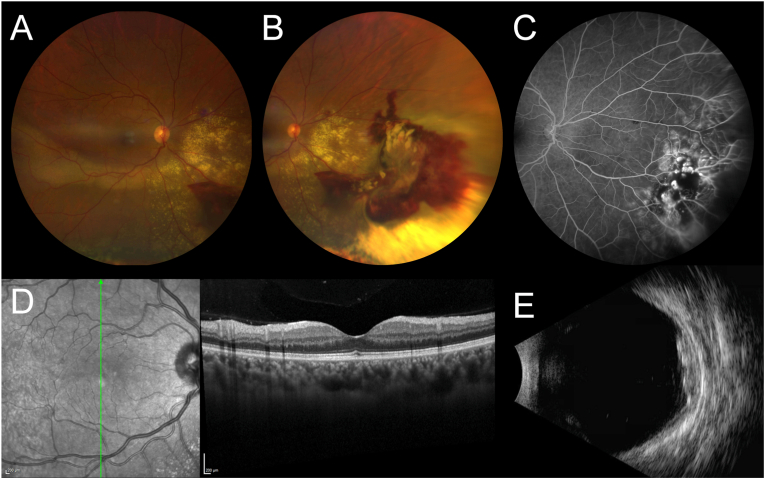
On presentation, fundus photos of the right eye showed (A) an exudative process involving the inferior macula with (B) a lesion in the inferonasal periphery consisting of arteriovenous nicking, arteriolar narrowing, “light-bulb”-appearing aneurysms, telangiectatic vessels, and significant exudation extending up to the optic nerve head with an exudative retinal detachment inferiorly. On fluorescein angiography, there was early and progressive perivascular leak, peripheral capillary non-perfusion, and capillary dropout in the right eye (C). Optical coherence tomography showed normal macular morphology (D). B-scan ultrasonography showed a retinal lesion with heterogenous echogenicity and low-to-medium internal reflectivity (E).

On presentation, her best corrected visual acuity (BCVA) was 20/20 in the right eye (OD) and 20/20 in the left eye (OS). Intraocular pressure (IOP) was 10 mmHg OD and 11 mmHg OS. Her anterior intraocular examination was normal. Dilated fundus exam (DFE) was notable for arteriovenous nicking, arteriolar narrowing, an inferonasal peripheral lesion with “light-bulb”-appearing aneurysms, telangiectatic vessels, and significant associated exudation extending up to the optic nerve head with an exudative retinal detachment (RD) inferiorly OD (Fig. 1A–B). The dilated fundus exam of OS was normal. On fluorescein angiography (FA), there was early and progressive perivascular leak, peripheral capillary non-perfusion, and capillary dropout and blockage OD (Fig. 1C) but was otherwise normal OS. Optical coherence tomography (OCT) of the macula showed normal macular morphology (Fig. 1D). B-scan ultrasonography showed a retinal lesion with maximal thickness of 2.7 millimeters (mm) with heterogenous echogenicity and low-to-medium internal reflectivity (Fig. 1E).

**Fig. 2:**
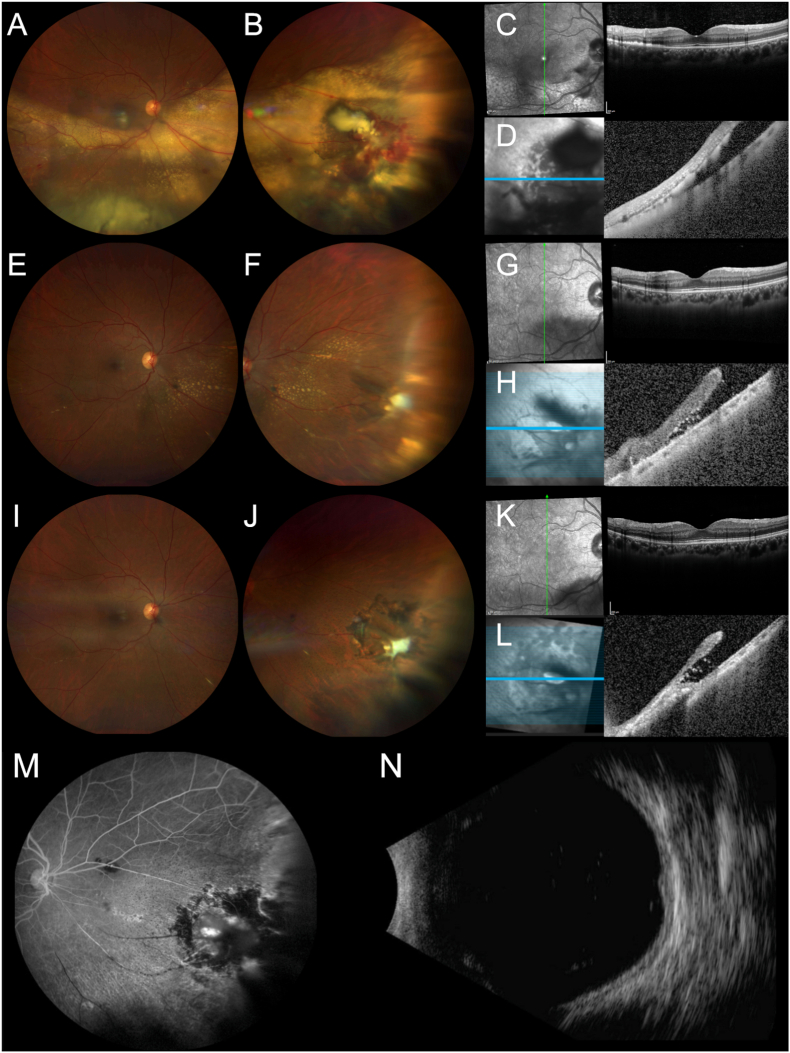
After three months of intravitreal aflibercept therapy, fundus photos of the right eye showed an obscured media with (A) a large exudative retinal detachment extending from the macula to the inferior periphery and (B) a large inferonasal peripheral lesion with hemorrhagic and exudative components most consistent with a retinal vasoproliferative tumor (RVPT). Optical coherence tomography (OCT) of the macula showed subretinal hyper-reflective material in the inferior macula with disruption of the ellipsoid zone (EZ) layer (C). OCT through the lesion showed retinal elevation with subretinal hyper-reflective material and fluid (D). After treatment with five intravitreal injections of faricimab-svoa, fundus photos of the right eye revealed significant improvement in the exudative retinal detachment with (E) almost complete resolution of exudation in the macula and (F) near resolution of the inferonasal hemorrhaging and exudation but the presence of an inferonasal retinal tear at the base of the RVPT with surrounding subretinal fluid. OCT of the macula showed resolution of the subretinal hyper-reflective material but residual EZ loss in the corresponding area (G). OCT through the lesion demonstrated a full thickness retinal break with subretinal hyper-reflective foci (H). Six months later, fundus photos revealed (I) stable complete resolution of exudation and hemorrhaging in the macula and periphery and (J) the presence of the prior inferonasal retinal tear with good laser barricade. OCT continued to show residual focal EZ loss in the inferior macula (K). OCT through the lesion also demonstrated a stable retinal break and interval retinal thinning (L). Late phase fluorescein angiography demonstrated staining of the regressed lesion (M). B-scan ultrasonography showed marked reduction of lesion thickness (N).

Exam and imaging findings were consistent with exudative retinopathy secondary to RVPT. The decision was to proceed with intravitreal anti-VEGF therapy. The patient received two additional intravitreal aflibercept treatments every 4 to 6 weeks. At her follow-up three months later, her BCVA was stable at 20/20 OU. However, DFE of OD showed vitreous hemorrhage over the inferior macula, and a large inferonasal lesion with hemorrhagic and exudative components with interval worsening compared to her initial visit (Fig. 2A–B). OCT of the macula showed subretinal hyper-reflective material in the inferior macula with disruption of the ellipsoid zone (EZ) layer (Fig. 2C). OCT through the lesion showed retinal elevation with subretinal hyper-reflective material and fluid (Fig. 2D). With the interval progression in exam findings, the decision was made to switch from aflibercept to faricimab injections given reports of the utility of faricimab in prior exudative retinal processes.^12^

After three rounds of monthly faricimab injections, exam of OD showed rapidly resolving vitreous hemorrhage and opacities, regression of the inferonasal lesion, and residual exudation. There was improvement in SRF underlying the lesion. Two additional monthly injections of faricimab were given due to residual exudation and SRF. Two months following the last faricimab injection, she was noted to have complete resolution of subretinal exudates and SRF and near complete regression of the RVPT but developed an inferonasal retinal tear at the base of the RVPT with surrounding SRF (Fig. 2E–F), likely due to rapid contraction of the lesion. OCT of the macula at this time showed resolution of the subretinal hyper-reflective material but residual EZ loss in the inferior macula (Fig. 2G). OCT through the lesion demonstrated a full thickness retinal break with subretinal hyper-reflective foci (Fig. 2H). She was treated with laser retinopexy and monitored off additional treatments.

Six months after completion of faricimab therapy, the patient's BCVA remained 20/20 OU. Her exam revealed stable complete resolution of the inferonasal hemorrhaging and exudation and the presence of the prior inferonasal retinal tear with good laser barricade (Fig. 2I–J). OCT continued to show residual focal EZ loss in the inferior macula (Fig. 2K). OCT through the lesion also demonstrated a stable retinal break and interval retinal thinning (Fig. 2L). FA demonstrated focal staining of the regressed lesion (Fig. 2M). B-scan ultrasonography showed a markedly reduced lesion thickness of 1.5 mm (Fig. 2N). At 1 year follow-up, her exam and imaging were stable with complete regression of the RVPT. Given the patient's clinical stability, she was monitored without further intervention.

## 3 Discussion

We present a case of intravitreal faricimab as a successful treatment of exudative retinopathy secondary to RVPT, which was refractory to intravitreal aflibercept. Faricimab treatment resulted in complete resolution of exudates and subretinal fluid, as well as regression of the RVPT without the requirement of additional cryotherapy or laser photocoagulation. This case highlights the use of intravitreal faricimab as an alternative therapy for patients with RVPT.

The treatment of RVPTs remains largely individualized, guided by size, location, and the degree of associated retinal damage. While ablative treatments such as cryotherapy and laser photocoagulation remain first-line options for large or active RVPTs, pharmacologic agents are increasingly being used to address secondary complications such as macular edema and exudation.^2^, ^4^

Anti-VEGF agents have been employed with varying success in the treatment of RVPTs. Prior reports have noted a reduction in tumor thickness in patients treated with intravitreal bevacizumab.^5^ However, intravitreal bevacizumab monotherapy has shown limited effectiveness in long-term regression of lesions, often requiring additional therapies including laser photocoagulation and/or cryotherapy to achieve complete regression.^5^^,^^6^ Intravitreal aflibercept monotherapy has shown efficacy in complete RVPT regression along with RVPT-associated exudative and neovascular changes, though in our case intravitreal aflibercept was not effective.^7^ Intravitreal ranibizumab in combination with laser photocoagulation therapy has also been noted to regress RVPT with good visual outcomes.^8^

Faricimab has emerged as a potent treatment option in retinal diseases characterized by both vascular leakage and inflammation. In the landmark nAMD (TENAYA and LUCERNE)^13^ and DME (YOSEMITE and RHINE)^14^ clinical trials, intravitreal faricimab demonstrated comparable or superior outcomes to aflibercept in visual acuity, anatomical improvements in edema, and central retina thickness. Faricimab's dual mechanism in inhibiting VEGF-A and Ang-2 has potential applicability in the treatment of RVPTs, both in addressing retinal exudation and edema. The pathogenesis of RVPTs involves a reactive proliferation of blood vessels due to retinal ischemia, inflammation, or injury with resulting vascular leakage and glial cell proliferation.^15^^,^^16^ Prior studies have elucidated that Ang-2 promotes vascular destabilization and sensitizes blood vessels to the effects of VEGF-A.^17^ By targeting both Ang-2 and VEGF-A, faricimab may inhibit the synergistic effects of these molecules in vascular leakage, neovascularization, and inflammation, thereby providing a mechanism of regressing exudative retinopathy and RVPTs that is unique compared to other classes of anti-VEGF therapies.

In our case, intravitreal faricimab was employed off-label to treat an exudative RVPT that was refractory to previous treatment with intravitreal aflibercept. The use of faricimab resulted in excellent anatomical and visual improvement, which lasted to 1 year follow-up. Of note in our case, there was the development of a retinal tear after five injections of faricimab that was successfully treated with laser retinopexy. We suspect that faricimab's efficacy in rapidly regressing the RVPT and exudation precipitated a tractional break in the retina.

Overall, faricimab may be beneficial as a primary treatment in patients with RVPTs. The use of faricimab as monotherapy or adjunctive therapy in RVPTs warrants further investigation in treatment trials with long-term follow up.

## CRediT authorship contribution statement

**Naveen Karthik:** Writing – review & editing, Writing – original draft, Visualization, Validation, Methodology, Investigation, Formal analysis, Data curation. **Syeda Fatima Abid:** Writing – original draft, Visualization, Validation, Investigation, Formal analysis, Data curation. **Yuxi Zheng:** Writing – review & editing, Visualization, Validation, Supervision, Investigation, Data curation, Conceptualization. **Miguel Materin:** Writing – review & editing, Visualization, Validation, Supervision, Investigation. **Xi Chen:** Writing – review & editing, Visualization, Validation, Supervision, Project administration, Investigation, Data curation, Conceptualization.

## Patient consent

Consent to publish this case report has been obtained from the patient in writing.

## Data statement

The data that support the findings of this study are available on reasonable request from the corresponding author, XC.

## Authorship

All authors attest that they meet the current ICMJE criteria for Authorship.

## Funding statement

This research did not receive any specific grant from funding agencies in the public, commercial, or not-for-profit sectors.

## Declaration of competing interest

The authors declare that they have no known competing financial interests or personal relationships that could have appeared to influence the work reported in this paper.
